# The Physician’s Personality and Its Influence1Read before the Roswell Park Medical Club, January 29, 1894.

**Published:** 1894-05

**Authors:** J. C. Thompson

**Affiliations:** 243 Dearborn Street; Buffalo, N. Y.


Buffalo Medical and Surgical Journal

Vol. XXXIII. MAY, 1894. No. 10.

### Original Communications.

### THE PHYSICIAN'S PERSONALITY AND ITS INFLUENCE.[1]

By J. C. THOMPSON, M. D., Buffalo, N. Y.

Physiological psychology is comparatively a new science. The scientific study of the soul, and especially the use of physical instruments of precision in the investigation of psychical attributes, has only just begun. Consequently, the influence exerted by this important part of our nature is, as yet, by many but faintly appreciated and by many more even less understood.

It is not meant to include in this essay a consideration of the physician's personality in relation to all psychical attributes, but rather the influence of personality on certain elements of the soul, represented by those subtle attributes and imponderable psychic qualities commonly termed the emotions.

The emotions influence the mind and modify organic function much oftener and more profoundly than superficial observation would cause us to suspect. It is their action which will quicken the heart-beats or arrest that vital organ in its function, influence the vaso-motors with the rapidity of thought so that the cheek will be suffused in shame or blanched in fear, make a man bold to the verge of rashness or a coward for conscience sake, cause the mother to defend her young with her life and, from love, endure any privation and affliction for the object of her affection.

The emotional faculty of the soul influences the mind, and thus becomes the basis or motive of physical activity to a greater extent than most individuals may be willing to acknowledge. For instance, in the case of children, it cannot be that their likes and dislikes are the result of reasoning or carefully drawn conclusions, because their intellectual faculties are as yet but in the dawn of

1. Read before the Roswell Park Medical Club, January 29, 1894.

their development. Their standard, if standard it may be called, is simply that of the instinctive action of their emotional nature, the product of their psychical feeling, which, in a measure, like instinct in the lower animals, is congenitally mature.

It has been said, and truly, "that adults" (and I add, with all due reverence for the sex, including women,) "are but children older grown." We have in this truism an easy explanation of certain facts observed by you all, viz., the common existence among adults of the most unreasonable likes and dislikes, incongruous marriages, erratic love affairs, and the strange actions of people usually denominated cranks, but who really are ordinary individuals misgoverned by their emotions instead of being governed by reason.

Probably no class of men see more of this mal-attempt at subjection of the human body than physicians. So true is this, that it becomes a part of their duty to study and administer to a diseased mind and its erratic emotions as often, perhaps, as they are called upon to treat a disordered body. Indeed, so important a rôle does the emotional faculty play in all physical affections that often actual lesions, distinct objective and subjective symptoms, may be caused by this subtle agency. In these cases, where there is apparently something substantially the matter, physicians are most liable to err. In their hard-headed search after a tangible cause for disordered function and actual suffering, they will pounce upon some slight deviation from the physical norm, and treat it externally, internally and eternally, without the slightest benefit to the poor patient, unless some suggestion or operation can, through a mental impression, rouse the nervous system to a more healthy action.

A case which admirably illustrates this point was, in a late journal, related by Goodell as follows:

Some years ago, a healthy lady, aged twenty-three, was engaged to a gentleman whom she tenderly loved. The wedding day was named, the clergyman was notified, and the invitations were sent out. Three days before the one fixed for the marriage there came a letter from the man, breaking off the engagement. The blow to her love and pride was, of course, great. She secluded herself in her room and in a few days took to her bed with many symptoms of nerve-prostration, of which dysuria and an irritable bladder were the most prominent. For these vesical symptoms she was treated by the family physician with external and internal electricity and with repeated flushings out of the bladder. Getting no better, but steadily worse, the next year she fell into the hands of a specialist, who diagnosticated anteflexion of the womb, cervical erosion, and fissure at the neck of the bladder. He dilated the urethra, washed out the bladder and treated the womb *secundem artem.* Still remaining an invalid, she consulted a third physician six months later. He also dilated the urethra, examined the bladder with an endoscope, washed it out repeatedly, applied electricity to it by an internal electrode and gave cold douches. No improvement following this treatment, she called in a fourth physician. He kept her for several weeks under his care, dilated the urethra, and treated her mainly with electricity, if I remember correctly, but of this fact I am not absolutely sure. At any rate, she was not made a whit better, and a fifth physician was summoned. He, without success, treated her for anteversion of the womb and rectal ulcer. She now saw her sixth physician. By this time her urethra had been dilated so frequently that, in the examination, his finger entered the meatus urinarius, and, not at first recognizing his mistake, he remarked upon the narrowness of the vagina. He recommended the formation of a button-hole fistula in the urethra, but his advice was rejected. Four years after the beginning of her disorder, she returned to the second physician whom she had previously consulted. After treating her heroically for some time, without the slightest benefit, he proposed the excision of the bladder. This operation was refused, and she, one year later, drifted back to her sixth physician. He first made a button-hole fistula in the urethra. This failing to do any good, an artificial vesico-vaginal fistula was next tried. No benefit accruing from this operation, her ovaries, as a last resort, were removed, but peritonitis set in and she died. Now, what is the true interpretation of this sad case? The terrible shock to her pride and to her affections shattered her nervous system, and she suffered more from a sore brain than from a sore bladder or from sore ovaries.

To go a step further, we are appalled to contemplate the extent to which faith, hope and religion are dependent upon the exercise and activity of the psychical faculty of emotion. It was the erratic emotion of one mind, Peter the Hermit, which started the crusades to rescue the Holy Sepulcher; in one of which, over 200,000 men, women and children participated, and all perished. Today the same emotional impulse sends hordes of weary pilgrims, by divers paths, to the sacred city of Mecca, where they imagine atoning grace can alone be found. In our lands and time have we not our Christian scientists and faith cures? And, alas! how many pseudo-holy creeds and opposite isms do the unthinking faithful follow and worship; impelled by the same emotional impulse which sent the hordes of central Europe to miserably perish on the burning sands of the desert.

Much more might be adduced to prove and illustrate the extent of psychic influence over human action, but a time limit compels haste to a consideration of the second phase of this subject: The influence of the physician's personality on this very important cause of disease. In this field of our warfare with affliction we are left without a tangible weapon. Our great standby (and I say it with shame), drugs, are here entirely useless. The most potent, or the most innocuous of them are equally unavailing in a full-fledged case of nervous disease from emotional origin. We can, with knife and pill, fight the demon month in and year out, till some poor fool of a faith doctor comes along, and presto! with one prayer, the patient is well. Now, why did not the regular physician offer up that prayer? And that is just what he should have done; not in so many words, but by so impressing the patient with his personality that there would have been no occasion for verbal prayer.

Perhaps you ask, about this time, what is meant by the physician's personality? Perhaps that is best answered by saying what it is not. A famous connoisseur in stringed instruments and antiques was asked wherein the renowned violins of Cremona, Stradivarius and Stainer possessed their super-excellence. He replied:

> It is not altogether in the well-selected and evenly-grained wood of which they are constructed, although that has something to do with it. It is not all in their incomparable shape and graceful swell, although that has something to do with it. Not all in the size and position of the openings or sound-holes, which also have their share in creating excellency. Not all in the quality and evenness of the varnish, but that, too, has its influence. It is all these and many other points combined that make the matchless and unrivaled qualities of the master violins.

So too, gentlemen, and I wish you to note this well, it is not the physician's personal appearance which makes his personality. It is not the charm of a well-modulated and resonant voice; not all in a graceful, hopeful walk and general carriage, although these all have to do with it. It does not all consist in personal cleanliness, good grooming and neatness of dress, but these are strong factors in it. Not all in undue familiarity, nor unwarranted coldness and austerity; not in overslopping sympathy nor frigid indifference, yet these qualities properly modified help much to create that positive, but incomprehensible and imponderable something, which for convenience and the lack of a better name we call animal magnetism, personal attraction, and the like.

It is that assemblage of qualities which creates in the minds of patients and others, respect, and even reverence, hope, ambition, faith, trustfulness; that which leaves a remembrance of the doctor's visit as a glorious, vivifying burst of sunshine, in the gloom and darkness of suffering and distress. Time permitting, endless well-authenticated cases might be cited, and countless authors referred to, to prove the correctness of this statement that the physician's personality acting on and through the patient's mind, always does much and often more to restore health than anything else. All possess this attribute, but in a varying degree—varying within a wide range. It may be cultivated by a painstaking practice of the many factors which serve to give it expression. For instance, if a man is habitually careless in his attire and personal appearance, hasty, excitable, erratic, untidy, he may improve all these. But he who by Nature is endowed with but a modicum of this physical attribute, is at a great disadvantage in the work of our guild.

In the cultivation of this power, remember that no counterfeit of interest, no false note of sympathy will avail, or pass for the genuine coin. Nowhere will more certainly be taken a correct inventory of genuine worth than by the exercise of this extraordinary sense in others; arriving at conclusions not by reason or logic, but by an intuition, true to fact as the needle to the pole. The great field for the exercise of the physician's personality, is with women, for in them the emotional nature is, in many instances, developed to the dignity of a special sense. In men this phase of the psychical is not so prominent, yet even here it plays no insignificant part in the estimation of character. In conclusion, the question arises, can we, as rational physicians, holding fast to that which is good, and constantly casting about for new weapons with which to combat disease, suffering and death, can we, I say, ignore an agent so potent for good, without which the most concentrated alkaloids may manifest their physiological action, but fail to produce the desired therapeutical effect?

Goodell, in the same article already quoted from, in trying to impress the lesson learned from meddlesome gynecology, says:

So misleading, indeed, are the symptoms of a jaded brain or other nerve strain, under the uterine guise in which they often masquerade, that when a jilted girl, a bereaved mother or a grieving wife consults a physician, he, unless on his guard, will be more likely to minister to a womb diseased than to a mind diseased. Such cases, even when associated with actual uterine disease, are not bettered by a merely local treatment, nor are medicines by themselves of much avail. What they need are the incantations of the rest-cure—namely, Massage, electricity and strict seclusion.

Hope should be infused into every case, and above all, mark you from Goodell, there should be imported into it the personality of the physician.

For myself, the longer and closer I study and observe the effect of the psychical over the physical, of the emotions over the body, the greater is my respect for this wonderful power, and the less am I inclined to doubt the remarkable tales of the utter subjection of the body to the soul, even to the suspension of animation for an indefinite period.

Cultivate a personality that will rob the faith-cures of their victims, and give to the healing art the true meed of praise for which she ought to strive—namely, that of being in the van of all true progress.

243 Dearborn Street.

